# Influence of standard culture conditions and effect of oleoresin from the microalga *Haematococcus pluvialis* on splenic cells from healthy Balb/c mice — a pilot study

**DOI:** 10.1007/s11626-023-00822-x

**Published:** 2023-12-07

**Authors:** Zuzana Jurčacková, Denisa Ciglanová, Dagmar Mudroňová, Daniela Bárcenas-Pérez, José Cheel, Gabriela Hrčková

**Affiliations:** 1 Institute of Parasitology, The Slovak Academy of Sciences, Hlinkova 3, 04001 Košice, Slovakia; 2 Department of Microbiology and Immunology, University of Veterinary Medicine and Pharmacy, Komenského 68, 04181 Košice, Slovakia; 3 Laboratory of Algal Biotechnology - Centre ALGATECH, Institute of Microbiology of the Czech Academy of Sciences, Opatovický Mlýn, 37981 Třeboň, Czech Republic; 4 Faculty of Science, University of South Bohemia, Branišovská, 1760, 37005 České Budějovice, Czech Republic

**Keywords:** Standard culture conditions, *Heamatococcus pluvialis*, Oleoresin, Oxidative stress, Mouse spleen cells

## Abstract

In this work, we used splenocytes from healthy mice to study the effects of the two most commonly used cell culture media (A, B) with different compositions of redox reagents. The incubation of cells for 24 h resulted in a significant decrease in viability and metabolic activity of splenocytes, and the negative effects of incubation in medium B were more pronounced. In standard conditions, oxidative stress in cells was manifested by reduced mitochondrial potential, and this effect correlated with the transition of 58.3% of cells to the early stage of apoptosis under reducing conditions of medium A and up to 66.1% of cells under super-reducing conditions in medium B, suggesting altered cell physiology. High levels of ROS/RNS activated transcription factor Nrf2, superoxide dismutase 1, and catalase. The higher mRNA levels of these genes were under the conditions of medium B, whose super-reducing environment in combination with the environment of conventional incubators proved to be less suitable for the cells compared to medium A. Treatment of the cells with a lower concentration (10 µg/ml) of oleoresin obtained from the microalga *H. pluvialis* partially eliminated the negative effects of cultivation. Higher concentration of oleoresin (40 µg/ml) was slightly cytotoxic, due to the significant antioxidant effect of astaxanthin, the main bioactive component of the extract, which eliminated most of the ROS/RNS acting as signalling molecules. This study shows that the standard culture conditions do not reflect the physiological *in vivo* cell conditions; therefore, they are not generally suitable for incubation of all cell types.

**Supplementary Information:**

The online version contains supplementary material available at 10.1007/s11626-023-00822-x.

## Introduction

Cell culture is a basic laboratory technique in which cells are grown under strictly controlled conditions outside their natural environment in a specific medium that supports their growth. The conditions usually vary depending on the cell type. Generally, cells are incubated in a culture medium that provides them with basic nutrients while regulating the physicochemical environment. This includes not only a constant temperature, but also a controlled osmotic pressure, pH, humidity, and oxygen flow (Salauddin 2022). The composition of the medium only affects the survival of the cultured cells, but can alter the proliferation of certain cell types, so the characteristics of the medium can directly affect research results (Vis *et al.* 2020).

The classical incubators with inlet of carbon dioxide (CO_2_) are used to ensure suitable cultivation conditions for cell cultures. CO_2_ offers many advantages in cultivation; most importantly, it provides optimal heat, sterile environment, humidity, and physiological pH (7.2–7.4) in the system. A bicarbonate-based buffer system is used to maintain a stable physiological pH in the culture medium containing cells (Maniarasu 2022). The principle is to dissolve CO_2_ in the medium, where it partially reacts with water and NaHCO_3_ to form carbonic acid, which in turn interacts with bicarbonate ions in the medium. The amount of CO_2_ is adjusted with sodium bicarbonate (NaHCO_3_) in the culture medium used. HEPES is commonly used to control pH in *in vitro* cultures, but the bicarbonate buffer system is more widely used due to its natural occurrence in the organism. 2-Mercaptoethanol (2-ME) is a strong reducing agent normally used in cell culture media to stabilize them by absorbing reactive molecules and preventing oxidation of unstable compounds (Bannai 1992). These conditions are essential for maintaining cell viability within at least 24 h. Oxygen is also an important factor in the cultivation of cells, significantly affecting the monitoring values of cell cultures. Oxygen is part of the gas phase of the culture medium. Under *in vivo* conditions, the O_2_ content in the extracellular space ranges from 2 to 14% depending on the tissue, which depends on the rate of blood flow through the capillaries and metabolic activity (Jagannathan *et al.* 2016; Mas-Bargues *et al.* 2019). Such conditions are referred to as physioxia or physiological normoxia. After oxygen is inhaled and delivered to various organs of the body, oxygen levels gradually decrease. However, cells are generally cultured *in vitro* under 18–21% of atmospheric oxygen, which is 2–5 times higher than in an *in vivo* environment (Atkuri *et al.* 2005, 2007). Several studies have shown that culturing cells at lower, more physiologically relevant oxygen tension (physioxia) has a positive effect on cell survival and proliferation (Carreau *et al.* 2011; Alva *et al.* 2022). Increased oxygen concentrations lead to increased production of reactive oxygen species (ROS), which has a significant effect on the physiology of the cultured cells (Halliwell 2003) and can potentially affect the results of the experiment itself.

Low (physiological) levels of reactive oxygen and nitrogen species are constantly produced in cells, and the redox balance is controlled by endogenous or exogenous antioxidant systems. Mitochondria play an important role in maintaining redox balance and the structural and functional integrity of cells. Antioxidant systems in the cells, including catalase, superoxide dismutase (SOD), lactoperoxidase, glutation, and glutathione peroxidase, are responsible for neutralizing the excess of ROS (Sztretye 2019). In the presence of excessive oxidative stress, the cell’s endogenous antioxidant systems may not be sufficient to maintain redox balance, and it is necessary to supply antioxidants from external sources.

In recent decades, carotenoids have attracted particular interest mainly because of their potent antioxidant, antiproliferative, anti-inflammatory, and reparative effects. Astaxanthin (3,3′-dihydroxy-β,β′-carotene-4,4′-dione) belongs to the xanthophyll carotenoid group and is one of the natural compounds with the strongest antioxidant activity (Park *et al.* 2010; Kim and Kim 2018; Sztretye 2019). Currently, more than 95% of astaxanthin available in the global market is synthetically produced, while natural astaxanthin is derived from the main natural source, the red form of the microalgae *Haematococcus pluvialis.* Astaxanthin is present in microalgae oleoresin, which is obtained by extraction with CO_2_ or organic solvents (Koller *et al.* 2014; Shah *et al.* 2016; Fabryova *et al.* 2020). Moreover, of this oleoresin, only 4–5% is free astaxanthin. The majority of astaxanthin of the oleoresin consists of esterified forms such as astaxanthin monoesters and diesters (Rao *et al.* 2015). Oleoresin also contains a minimal amount of other carotenoids, particularly lutein, canthaxanthin, and β-carotene. Synthetic astaxanthin, produced from Asta-C15-triarylphosphonium salt and C10-dialdehyde via the Wittig reaction (Krause *et al.* 1997), has 20-fold lower antioxidant capacity than its natural analogue and is not yet approved for human consumption (Koller *et al.* 2014; Lorenz and Cysewski 2000). In addition, there are concerns about the safety of using synthetic astaxanthin for direct human consumption due to differences in stereochemistry and the potential carryover of synthetic intermediates. Because of these concerns, the natural oleoresin rich in astaxanthin from *H. pluvialis* is the preferred choice for pharmacological and nutritional applications (Li *et al.* 2011).

In our study, we first evaluated the effects of oxidative stress induced in mouse spleen cells by supraphysiological levels of atmospheric oxygen in culture conditions and the contribution of reducing conditions to ROS. In addition, we investigated the biological functions of spleen cells cultured in two media with different ROS-reducing capacity after exposure to oleoresin. We examined the effects on viability, ROS/NO production, and antioxidant gene expression to demonstrate the importance of proper culture conditions for testing the antioxidant effects of natural compounds such as astaxanthin.

## Materials and methods

### Chemicals and reagents

Two types of cell culture media were used for incubation of splenocytes during experimental procedures. Medium A was RPMI 1640 (Biochrom-Merck, Darmstadt, Germany) without sodium bicarbonate, with phenol red, 2 mM stable glutamine, and supplemented with 10% heat-inactivated foetal bovine serum (Biochrom, Berlin, Germany), 100 U/ml penicillin, 100 μg/ml streptomycin, 10 μg/ml gentamicin, and 2.5 μg/ml amphotericin B (all from Sigma-Aldrich, St. Louis, MO) were used. The second medium tested, designated medium B, was RPMI 1640 medium (Lonza, Verviers, Belgium) containing 25 mM sodium bicarbonate, 2 mM stable glutamine and phenol red was supplemented with 50 μM 2-ME (Gibco, New York, NY), 10% heat-inactivated foetal bovine serum, 100 U/ml penicillin, 100 μg/ml streptomycin, 10 μg/ml gentamicin, and 2.5 μg/ml amphotericin B.

The other chemicals used for the assays were 3-(4,5-dimethylthiazol-2-yl)-2,5-diphenyltetrazolium bromide (MTT, AppliChem, Darmstadt, Germany), Trypan blue solution (Biochrom, Berlin, Germany), Dimethyl sulfoxide (DMSO, Invitrogen/Thermo Fisher Scientific, Carlsbad, CA), 2′,7′-dichlorodihydrofluorescein diacetate) (H_2_DCFDA, Invitrogen, Carlsbad, CA), Neutral Red, 2,2′-azo-bis-(2-methylpropionamidine) dihydrochloride (AAPH) 1 M stock solution, 123-Rhodamine. The Griess reagent was prepared from 5% orthophosphoric acid, 1% sulphanilamide, and 0.1% N-(1-Naphthyl) ethylenediamine dihydrochloride standard was NaNO_2_ (the following chemicals were purchased from Sigma-Aldrich, St. Louis, MO).

Other chemicals including NH_4_Cl, EDTA, and sodium azide were used for cell biology applications. Methanol with purity of 99.99% (HiPerSolv, Chromanorm, Fontenay-sous-Bois, France) from VWR was used for HPLC analysis of astaxanthins. *H. pluvialis* biomass was purchased from Algamo, s. r. o. (Mostek, Czech Republic).

### Preparation of oleoresin from *Haematococcus pluvialis*

For the preparation of oleoresin from *H. pluvialis*, 60 g of dry biomass of the microalga was used, which was extracted with 600 ml of acetone. The extraction process was assisted by sonication with an ultrasonic bath (K6 Kraintek, s.r.o., Podhájska, Slovakia) with a frequency of 38 kHz and an intensity of 47.7707 W/cm at 25°C and for 30 min. The resulting suspension was centrifuged to facilitate the removal of suspended particles. The extraction procedure was repeated three times with the residual biomass. The supernatant obtained from the centrifugation of the extraction procedures was combined and evaporated under reduced pressure using a rotary evaporator at a temperature of 30°C. 22.05 g of oleoresin was obtained, which was used for biological evaluation.

### HPLC-APCI-HRMS analysis of the oleoresin from *H. pluvialis*

Analysis of *H. pluvialis* oleoresin was performed using a Dionex UltiMate 3000 HPLC system (Thermo Scientific, Sunnyvale, CA) coupled to a high-resolution tandem mass spectrometry (HRMS/MS) detector with atmospheric pressure chemical ionization (APCI) source (Impact HD mass spectrometer Bruker, Billerica, MA) (HPLC-APCI-HRMS). A Luna® C8 column (100 × 4.6 mm, 3 μm, Phenomenex) maintained at a temperature of 30°C was used to separate the target compounds. The mobile phase consisted of water (A) and methanol (B), both with 0.1% formic acid to increase ionization efficiency. An elution gradient of the mobile phase with a flow rate of 0.8 ml/min was applied as follows: 0–20 min, 20–0% A; 20–25 min, 0% A; 25–27 min, 0–20% A; 27–30 min, 20–20% A. The conditions for MS analysis were as follows: dry temperature 250°C; drying gas flow 12 l/min; nebulizer 3 bar; the voltage of the spray needle 4.2 kV. Mass spectra were recorded in positive ionization mode in the range of 50–2000 m/*z* at a scanning rate of 2 Hz. Collision energy of 35 eV was used, with nitrogen serving as the collision gas. Sodium formate clusters were used for spectral calibration at the beginning of each analysis. The Smart Formula function in Bruker Compass DataAnalysis software (version 4.2) was used to calculate the formulas for the molecular peaks and fragments. Identification of individual oleoresin peaks was determined by comparing data from HPLC-APCI-HRMS with existing literature data. The HPLC analysis of oleoresin is shown in Supplementary Fig. 1.

### Isolation and cultivation of mouse spleen cells

Male and female Balb/c mice were originally purchased from Velaz (Prague, Czech Republic) and bred in at the animal facilities of Institute of Parasitology of the Slovak Academy of Sciences under pathogen-free conditions. Spleens were aseptically isolated from 2 to 3 male mice in each experiment. Cell suspensions were obtained by passing spleen tissue through 40 μm nylon filters (BD Biosciences, Darmstadt, Germany) in 5 ml of cold culture medium A. Red blood cells were removed by incubating the suspension with 5 ml of lysis solution (8.02% NH_4_Cl, 0.85% NaHCO_3_, and 0.37% EDTA) for 5 min on ice. After centrifugation, splenocytes were washed twice in PBS and filtered through nylon filters (BD Biosciences). Mouse splenocyte suspensions were pooled, counted, and analysed for viability using the trypan blue exclusion assay. Then, the cells were resuspended in medium A or medium B for the experiments. The study was performed in accordance with the Guidelines for the Care and Use of Experimental Animals, No. 289/2003, in the Slovak Republic. The experiment was approved by the Ethics Committee of the State Veterinary and Food Administration of the Slovak Republic.

### Experimental design

**Figure 1.**
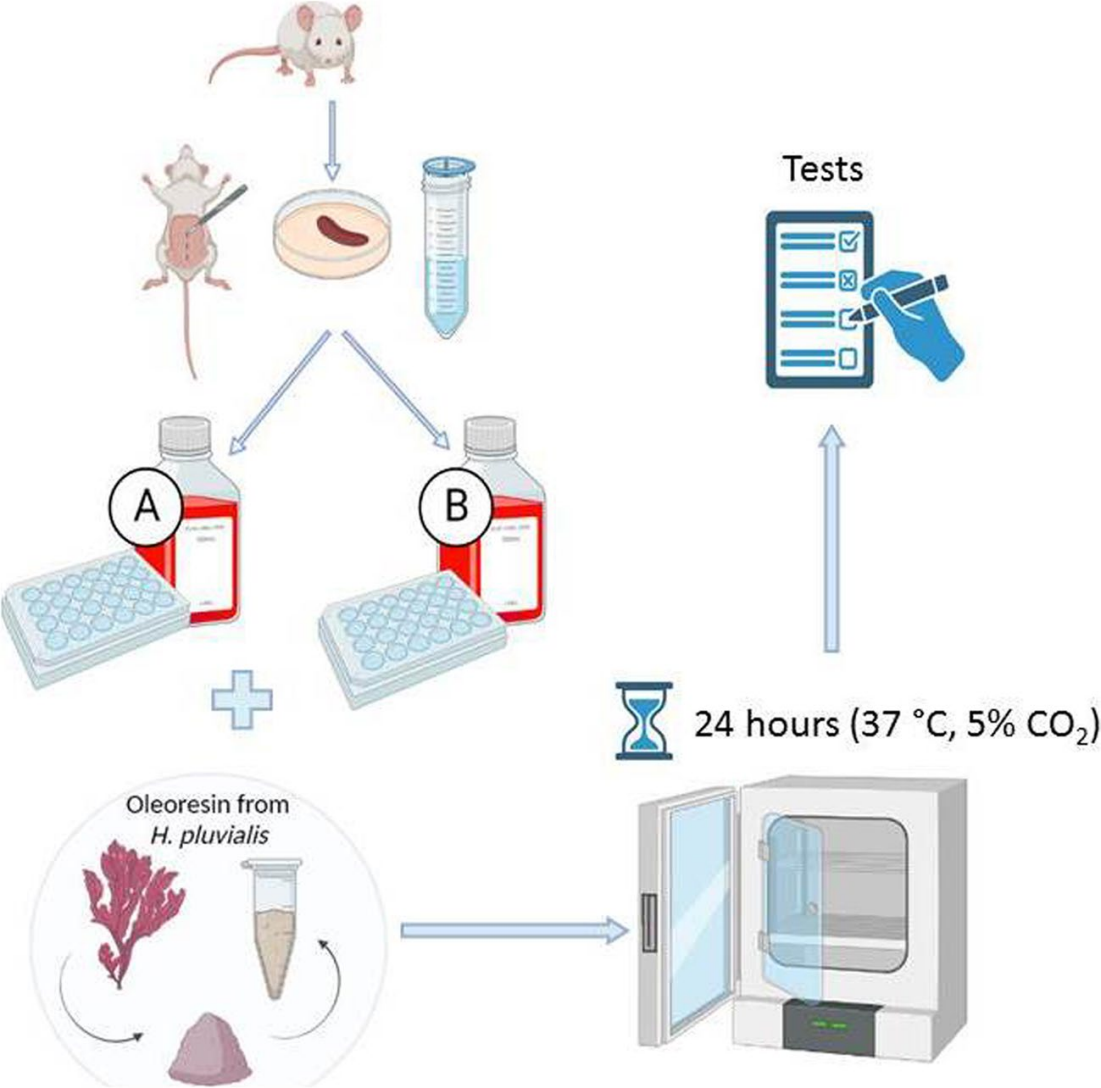
Created with BioRender.com

In order to obtain a first insight whether standard culture conditions can affect splenocyte viability after 24 h of incubation, an experiment was designed using cells cultured in two media that differ in free radical reduction conditions (Fig. 1). In addition, oxidative stress was induced in the cells by a water-soluble molecule 2,2′-azo-bis-(2-methylpropionamidine) dihydrochloride (1 mM) for 1 h in the assays to monitor the level of ROS. The AAPH system stimulates the production of free radicals at physiological temperature without generating H_2_O_2_ as an intermediate, thereby achieving an independent effect from the enzymatic antioxidant system of cells. Within these experimental settings, we assessed viability, metabolic activity, neutral red absorption, mitochondrial membrane potential, expression of genes controlling endogenous antioxidant mechanisms, intracellular ROS production, and nitrite oxide production. To determine how the influence of culture conditions is modulated by the addition of oleoresin (OLR) with significant antioxidant effect, we also repeated all tests after addition of OLR to splenocytes in both types of culture media.

### Trypan blue exclusion (viability test)

Splenocyte suspension diluted to a concentration of 1 × 10^6^ cells/ml in medium (A or B) was placed on CultureSlides (Falcon Tissue Culture Treated Glass Slides, Corning, Glendale, AZ) in triplicate for each treatment. OLR working solution was added to the cells in a concentration-dependent manner. Splenocytes were incubated for 24 h at 37°C, 5% CO_2_, humidity, and atmospheric oxygen in medium A and B, respectively. Untreated cells in medium A and B were used as controls, and naïve, freshly isolated cells were used as intact controls. Trypan blue (10% solution, 10 µl) was added to the wells, and the proportions (%) of live cells to total cells/well counted (for a total of 300 cells) were calculated for the control sample and each OLR concentration. Final data are given as mean ± SD. Viability was assessed and verified in two independent *in vitro* experiments.

### MTT assay

The MTT assay is a colorimetric test for the evaluation of cellular metabolic activity based on the conversion of the water-soluble yellow dye MTT (3-(4,5-dimethylthiazol-2-yl)-2,5-diphenyltetrazolium bromide) to insoluble purple formazan by the action of mitochondrial and cytoplasmic reductases (Berridge and Tan 1993; Kumar *et al.* 2018). A suspension of splenocytes diluted to a concentration of 1 × 10^6^ cells/ml in medium (A or B) was placed in 24-well plates (Corning, Glendale, AZ) in triplicate for each treatment and control, and cells were then subsequently incubated for 24 h under the same conditions as described above. Untreated cells in both medium A and medium B were used as control and naïve, freshly isolated cells as intact control. OLR working solution was added to the cells in a concentration-dependent manner. MTT (5 mg/ml in PBS) was added to the cell suspension 4 h before end of the assay in a volume of 50 μl/ml. The non-adherent cells were transferred to tubes, and the adherent cell population was washed with PBS. Then, 100 µl of DMSO was added to each well and to the tubes containing the non-adherent cells. OD values for adherent and non-adherent cells/sample were summed and used to calculate the mean ± SD. The *in vitro* experiment was repeated three times for spleen cells cultured in both medium A and B.

### Neutral red uptake assay

The principle is the ability of living cells not only to incorporate, but also to bind the dye neutral red in response to the pH gradient through the production of ATP in lysosomes, and this after only a short period of co-cultivation (Repetto *et al.* 2008). A suspension of splenocytes diluted to a concentration of 1 × 10^6^ cells/ml in medium (A or B) was placed in 24-well plates in triplicate for each treatment and control. Untreated cells in medium A/B were used as control and naïve, freshly isolated cells as intact control. OLR at selected concentrations was added to the cells, and splenocytes were incubated for 24 h under standard conditions. One hour before the end of cultivation, 20 µg/10 µl of neutral red was added to each well. Dye extraction was performed separately for non-adherent and adherent cells using 200 μl of extraction buffer (1% glacial acetic acid, 50% ethanol in distilled water). Optical density of extracts from both cell counterparts/wells was measured in a 96-well plate at 550 nm using a Multiscan FC Plate Reader. Two *in vitro* experiments were performed.

### Mitochondrial membrane potential

Mitochondrial membrane potential reflects the process of electron transport and oxidative phosphorylation, which is why it is considered a key indicator of mitochondrial activity (Chao *et al.* 2017). Loss of mitochondrial potential indicates bioenergetic stress and subsequently leads to the release of apoptotic factors that result in cell death (Gutierrez *et al.* 2017). Splenocytes diluted to a concentration of 1 × 10^6^ cells/ml in medium A or B were treated with selected concentrations of OLR in 24-well plates for 24 h. Naive cells were used as intact control. Untreated cells in medium A or B were used as control. Mitochondrial membrane potential (Δψm) was determined by flow cytometry using the fluorescent dye rhodamine 123, which is absorbed by mitochondria of living cells. Changes in dye absorption reflect Δψm and are expressed as mean fluorescence intensity (MFI). After vigorous resuspension, splenocytes were transferred to test tubes and a dye was added at a final concentration of 10 µM. Cells were incubated at 37°C for 20 min, then centrifuged, the supernatant was removed, and cells resuspended in PBS were immediately used to measure Δψm. Flow cytometry was performed using a FACS Canto flow cytometer with rhodamine 123 excitation at 505 nm and emission at 535 nm. The experiment was performed three times.

### Annexin V/propidium iodide apoptosis assay

The apoptosis assay allows detection of changes in cells associated with programmed death and then quantification of cells undergoing apoptosis, which allowed us to evaluate the effects of the culture environment as well as the influence of the extract tested. Splenocytes were diluted to a concentration of 1 × 10^6^ cells/ml in medium A or medium B, plated in plates with 24 wells, and treated with OLR (10 and 40 µg/ml) for 24 h. Untreated cells were used as control and naïve cells as intact control. After the incubation period, the non-adherent splenocyte population was transferred to tubes, and the adherent cell population was detached with 300 µl of warm Accutase Cell Detachment Solution (BioLegend, San Diego, CA) for 20 min at 37°C, collected into tubes, and washed. Then, both cell fractions/wells were pooled, washed in cold PBS, and stained at room temperature with Annexin V and propidium iodide solutions using the BD Pharmingen Annexin V-FITC apoptosis detection kit (BD Biosciences, San Jose, CA) according to the manufacturer’s instructions. Analysis was performed by flow cytometry using a FACS Canto flow cytometer. The percentages (%) of live cells and cells in different stages of apoptosis were evaluated using FACS Diva software. The assay was performed twice.

### Determination of the amount of nitric oxide

Suspensions of splenocytes diluted to a concentration of 1 × 10^6^ cells/ml in medium A or B were plated in 24-well plates in triplicate/treatment types and treated with OLR (10 and 40 µg/ml) for 24 h. Untreated cells in medium A/B were used as control, and freshly isolated (naïve) cells were used as intact control group. Two hours before the end of cultivation, splenocytes were treated with AAPH (10 mM) to induce nitric oxide (NO) production. The concentration of NO in the supernatant was measured as nitrite (NO_2_^−^) using the Griess reagent in a 96-well plate (in triplicate). Absorbance was measured at 550 nm using a Multiscan FC Plate Reader. Nitrite concentration was determined from a calibration curve using 0.1 M NaNO_3_ as a standard.

### Determination of intracellular reactive oxygen species in splenocytes

Splenic cells diluted in medium A or medium B to a concentration of 1 × 10^6^/ml were plated in 24-well plates in triplicate/treatment and treated with OLR (10 and 40 µg/ml) for 24 h. Untreated cells in both medium A and B were used as control and naïve, freshly isolated cells as intact control. The production of intracellular reactive oxygen species was determined using the fluorescent dye H_2_DCFDA. The dye was added at a concentration of 1 mM 4 h before the end of incubation. To induce oxidative stress in cells, AAPH (10 mM) was added to cell cultures 1 h before the end of the assays. Non-adherent cells were transferred to tubes; adherent splenocytes were detached with 300 µl of warm Accutase for 20 min at 37°C and then transferred to tubes and washed. Both cell fractions from each individual well were pooled and resuspended in 200 µl of culture medium (A or B). The percentage of cells producing ROS and the mean fluorescence intensity (MFI) corresponding to the concentration of ROS were measured by flow cytometry using a FACS Canto flow cytometer. The assay was repeated twice for both culture media.

### RNA isolation from splenic cells and quantitative RT-PCR analysis

Cell suspensions (1 × 10^6^/ml) diluted in medium A or B were placed in 24-well plates (Corning, NY) in triplicate for each treatment and treated with 10 or 40 µg/ml OLR concentration for 24 h. Untreated cells in medium A and B were used as control. Naïve, freshly isolated cells were used as intact control. Supernatants containing lymphocytes were collected in 1.5-ml tubes, and cell pellets were immersed in 0.5 ml of Trizol reagent (Invitrogen). Adherent cells were immersed in 0.5 ml of Trizol, and both cell fractions/wells were pooled and used for RNA extraction. RNA was quantified using an AstraGene Nanospectrophotometer (Harston, Cambridge, UK), and then, 2 µg was transcribed into cDNA using ReverseAid H minus M-MuLV reverse transcriptase and oligodT primers (Thermo Fisher Scientific, Burlington, Canada). The cDNA for each sample was used as a template for quantitative PCR. RT-PCR analysis of relative mRNA abundance was determined using SYBR green master mix (Sigma-Aldrich, St. Louis, MO) on a BioRad CFX thermocycler (BioRad, Hercules, CA). RT-PCR was performed in 20 μl reactions. The list of primers from the study by Wang *et al.* (2019) used in qRT-PCR is summarized in Table 1. The *in vitro* experiment was repeated twice.

**Table 1.** Primers used for gene expression analysis by qRT-PCR

Target gene	Orientation	Sequence
GAPDH	Forward	5′-AGGTCGGTGTGAACGGATTTG-3′
	Reverse	5′-TGTAGACCATGTAGTTGAGGTCA-3′
Nrf2	Forward	5′-CTTTAGTCAGCGACAGAAGGAC-3′
	Reverse	5′-AGGCATCTTGTTTGGGAATGTG-3′
Superoxide dismutase	Forward	5′-AACCAGTTGTGTTGTCAGGAC-3′
	Reverse	5′-CCACCATGTTTCTTAGAGTGAGG-3′
Catalase	Forward	5′-AGCGACCAGATGAAGCAGTG-3′
	Reverse	5′-TCCGCTCTCTGTCAAAGTGTG-3′

### Statistical analysis

All data were calculated as means ± standard deviation (SD) from two or three independent *in vitro* experiments. Statistical analysis of data was performed using GraphPad Prism (version 7) for Windows (GraphPad Software, Inc., San Diego, CA). Results were analysed using either one-way analysis of variance (ANOVA) followed by Tukey’s post hoc test or grouped analysis using two-way ANOVA and Sidak post hoc test. Statistically significant differences were calculated for *p < 0.05, **p < 0.01, and ***p < 0.001.

## Results

### The effect of cultivation and *H. pluvialis* oleorein on splenocyte viability

**Figure 2.**
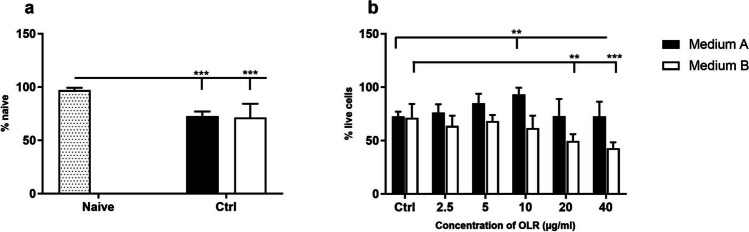
Effect of (*a*) culture conditions and (*b*) concentration-dependent effect of oleoresin from *H. pluvialis* — OLR on viability of splenocyte *in vitro* based on trypan blue absorbance in culture media A and B with different composition after 24 h. Significant changes in values compared to (*a*) naïve cells and (*b*) control group are indicated by **p < 0.01; ***p < 0.001; other values were not significant.

The trypan blue exclusion assay was used to determine the number of viable cells in a cell suspension. The effect of cultivation on spleen cell viability is shown in Fig. 2. Cultivation for 24 h had a negative effect on cell viability, as evidenced by a decrease in the percentage of viable spleen cells compared with naive, freshly isolated cells. To clarify the concentration-dependent effect of OLR on cell viability, spleen cells were cultured in two different compositions of culture media (Fig. 2). After culturing in medium A, no cytotoxic effect on the cells was observed at any OLR concentration. In contrast, a decrease in the viability of cells cultured in medium B was observed with increasing OLR concentration. Moreover, the viability of cells cultured in medium A with OLR concentration up to 10 µg/ml was significantly higher compared to cells in the control, and a decrease in the number of living cells was not observed in medium A even at low (2.5 µg/m) and high concentrations (40 µg/m).

### Effects of culture condition and *H. pluvialis* oleoresin on the metabolic activity of splenocytes evaluated by MTT test

**Figure 3.**
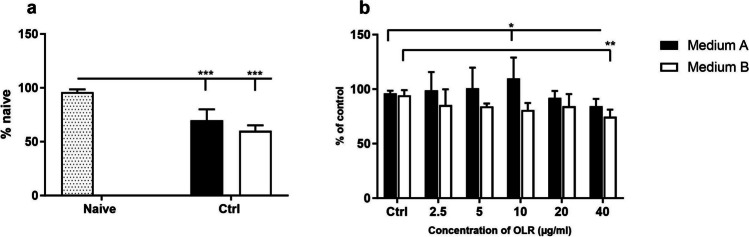
Effect of (*a*) culture conditions and (*b*) concentration-dependent effect of oleoresin from *H. pluvialis* — OLR on metabolic activity *in vitro* based on MTT assay in culture media A/B with different compositions after 24 h. Significant changes in values compared to (*a*) naïve cells and (*b*) control group are indicated by *p < 0.05; **p < 0.01; ***p < 0.001.

The MTT test is a colorimetric test that is commonly used to evaluate the metabolic activity of cells. We observed the monitored changes in metabolic activity of spleen cells cultured in two different compositions of culture media. Culturing in either medium significantly decreased cell metabolism, which was more suppressed in medium B compared with naive splenocytes (Fig. 3*a*). Subsequently, the metabolic activity of spleen cells cultured in two culture media was assessed after addition of OLR. Lower OLR concentrations (2.5 and 5 µg) increased the metabolic activity of splenocytes cultured in medium A, which was not observed for cells cultured in medium B. However, all monitored OLR concentrations decreased the metabolic activity of spleen cells cultured in medium B after 24 h of cultivation (Fig. 3*b*).

### The effects of culture condition and *H. pluvialis* oleoresin on splenocytes evaluated by neutral red uptake

**Figure 4.**
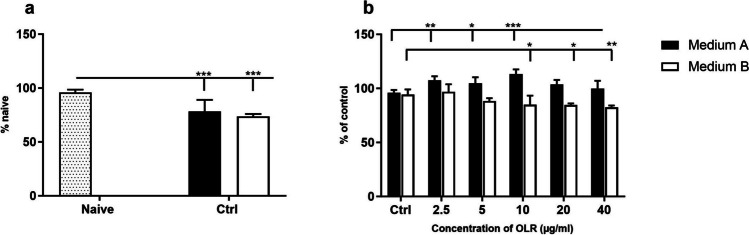
Effect of (*a*) culture conditions and (*b*) concentration-dependent effect of oleoresin from *H. pluvialis* — OLR on cytotoxicity of splenocytes *in vitro*, based on neutral red uptake assay in culture media A and B with different compositions after 24 h. Significant changes in values compared to (*a*) naïve cells and (*b*) control group are indicated by *p < 0.05; **p < 0.01; ***p < 0.001.

A neutral red uptake assay was also performed to quantitatively determine the number of live cells. Incubation of splenocytes for 24 h resulted in a significant decrease in the number of live cells in both media compared with naïve cells (Fig. 4*a*). Addition of OLR to cells increased splenocyte viability at all concentrations in medium A (*p* < 0.01 for 10 µg/ml). In contrast, addition of OLR to medium B resulted in a decrease in viability at all monitored concentrations. The greatest decrease in dye uptake by cells cultured with OLR in medium B was observed at a concentration of 40 µg/ml (Fig. 4*b*).

### Determination of intracellular ROS production

**Figure 5.**
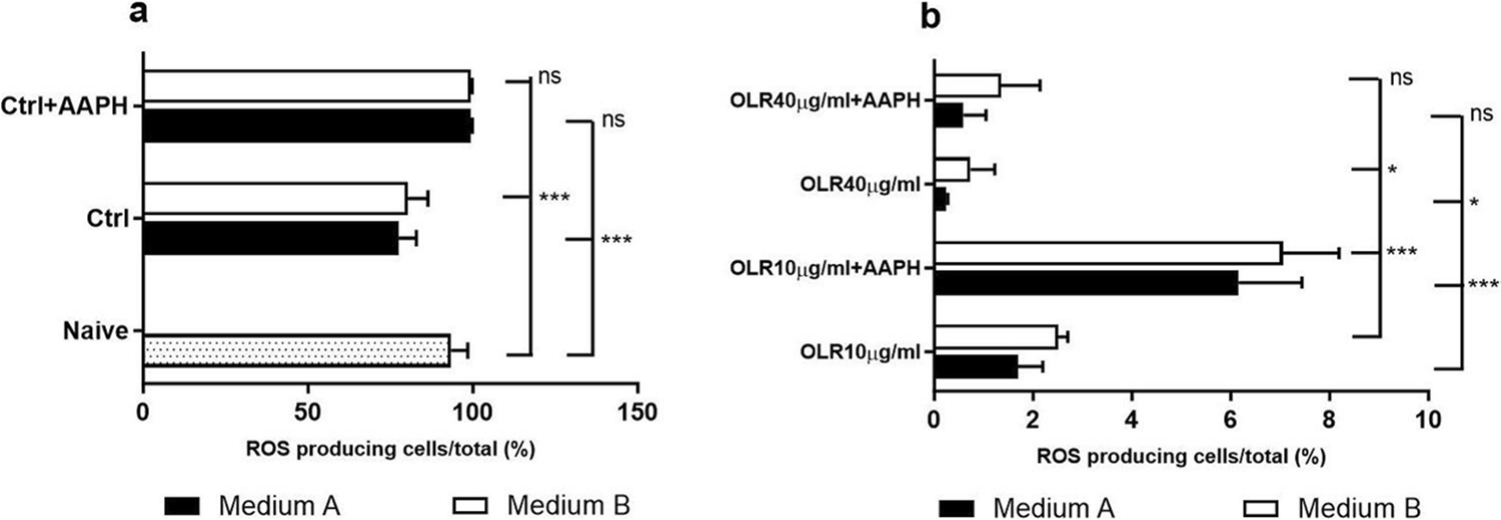
Intracellular production of reactive radicals in splenocytes after stimulation of radical production by 2,2′-azo-bis-(2-amidinopropane) dihydrochloride (AAPH). AAPH was added 1 h before the end of cultivation. The percentage of ROS-producing (*a*) naïve cells, (*b*) cells treated with ORL (10 and 40 µg/ml). Significantly different values are indicated by *p < 0.05; **p < 0.01; ***p < 0.00. ns, non-significant difference.

The majority of intracellular ROS is produced in mitochondrial respiratory chain in response to xenobiotics, cytokines, or bacterial infections, generating toxic metabolic by-products (Bae *et al.* 2011; Liu *et al.* 2020). When we examined the effects of culture conditions on the production of intracellular ROS, we found that the components contained in medium A and B decreased the production of free radicals compared with freshly isolated cells (Fig. 5*a*). The addition of OLR significantly decreased the production of reactive oxygen species, with a stronger effect observed at a higher concentration (40 µg/ml) of OLR in medium A compared to medium B (Fig. 5*b*).

### The effect of culture conditions and *H. pluvialis* oleoresin on apoptosis

**Figure 6.**
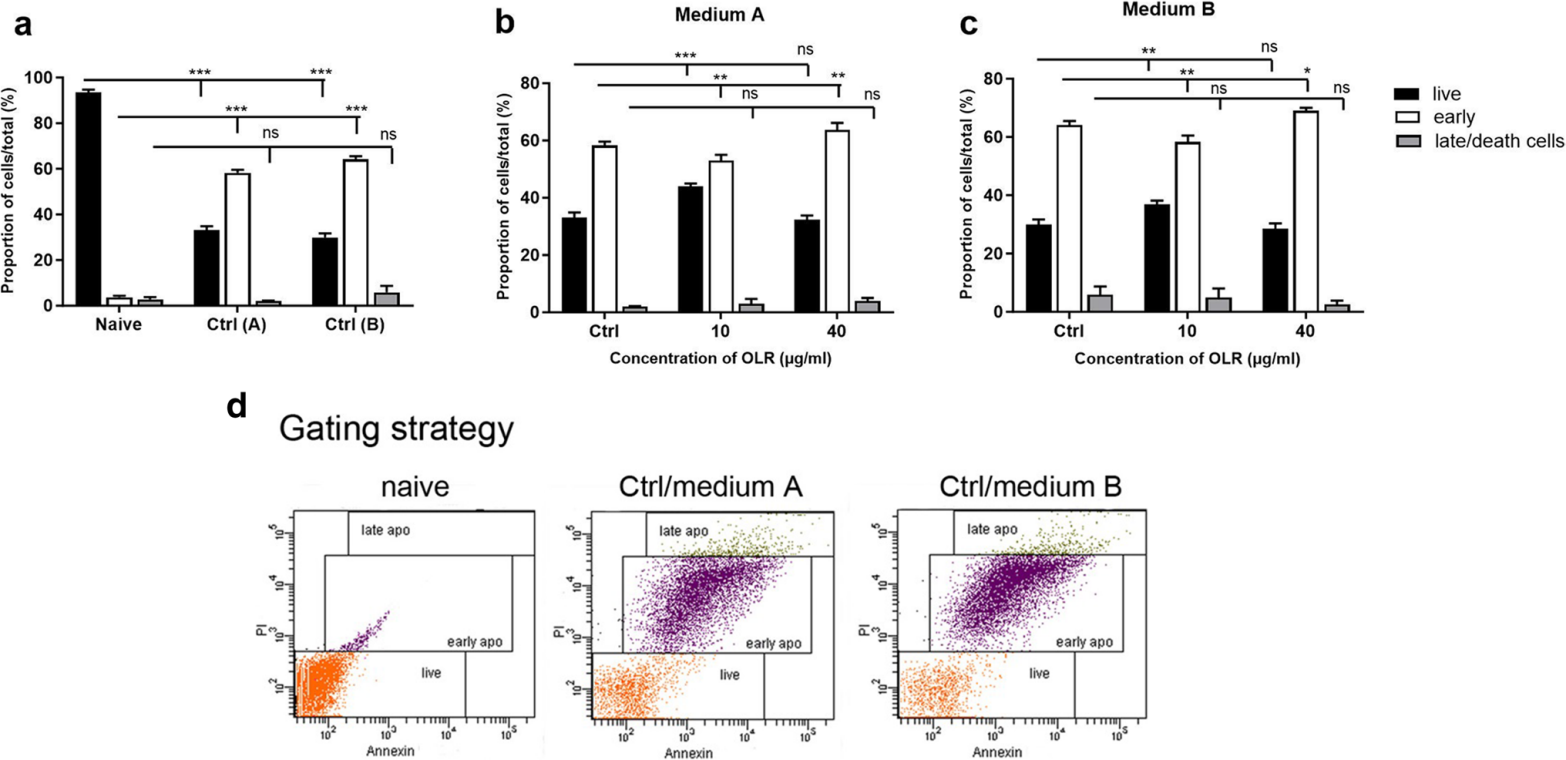
Effect of (*a*) culture conditions and (*b*, *c*) oleoresin from *H. pluvialis* — OLR (10 and 40 µg/ml) on the percentage of live cells, cells in the early phase of apoptosis, and cells in the late phase-dead cells in culture media A and B with different compositions after 24 h. Significant changes in the values in comparison with the naïve cells and the control group are indicated by **p < 0.01; ***p < 0.001. ns, not significant. (*d*) Gating strategy.

Apoptosis is a mechanism used to eliminate unnecessary and damaged cells through a series of morphological and biochemical changes in which the cells destroy themselves. Therefore, the regulation of apoptosis is essential for the proper functioning of cells and the maintenance of their homeostasis (Redza-Dutordoir and Averill-Bates 2016). We examined the effects of culture medium and OLR (10 and 40 µg/ml) on the percentage of live cells, cells in the early stage of apoptosis, and cells in the late stage of apoptosis/dead cells using flow cytometry. We also monitored apoptosis in naïve-freshly isolated cells and cells after 24 h of incubation (Ctrl). While most freshly isolated cells were live (93.4%) and only 3.7% were in the early stage of apoptosis (Fig. 6*a*), in the control group, only 33% of cells were live and up to 58.3% were in the early stage of apoptosis in medium A. In cells incubated in medium B, only 30% were live cells and 66.1% of cells were in the early stage of apoptosis (Fig. 6*b*). The addition of OLR (10 µg/ml) to the cells incubated in medium A mitigated the effects of the culture conditions and increased the percentage of live cells compared to the control group. Medium B supplemented with OLR at a concentration of 10 µg/ml also had a slight stimulatory effect on splenocytes, but the total number of live cells was lower in medium A. An OLR concentration of 40 µg/ml failed to prevent the induction of changes leading to apoptosis and resulted in a significantly higher proportion of cells in the early stages of apoptosis (63.6% in medium A; 69% in medium B) (Fig. 6*c*), correlating with a decrease in mitochondrial membrane potential.

### Analysis of mitochondrial membrane potential by flow cytometry

**Figure 7.**
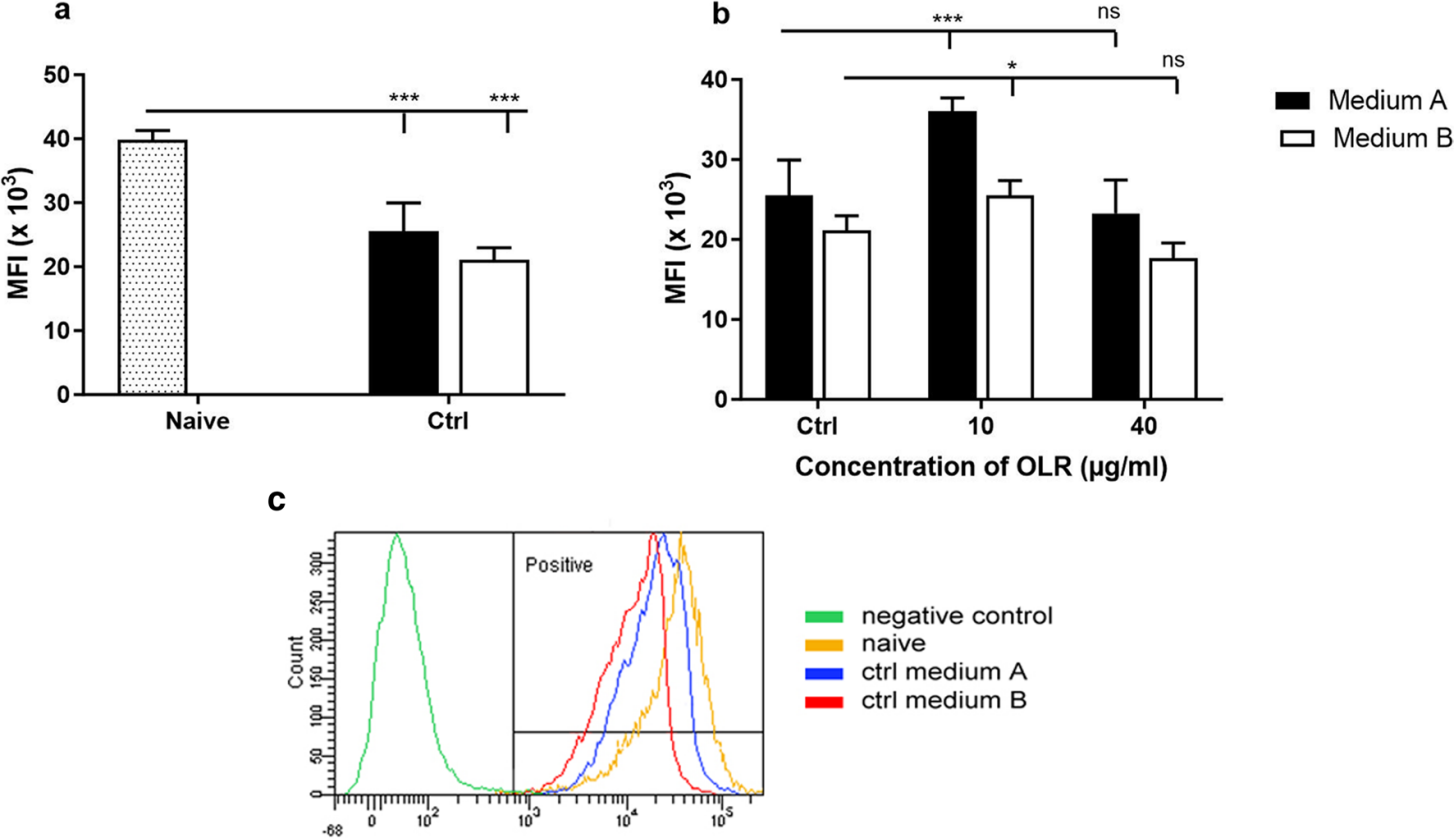
Effect of (*a*) culture conditions and (*b*) oleoresin from *H. pluvialis* — OLR (10 and 40 µg/ml) on spleen cells after 24 h of incubation in culture media A and B with different compositions based on changes in colour intake (ψm). Changes are expressed as mean fluorescence intensity (MIF) for rhodamine 123 absorbed by mitochondria of living cells. Significantly different values compared to (*a*) naïve cells and (*b*) control group are indicated by *p < 0.05; ***p < 0.001. (*c*) Gating strategy.

In our work, we further investigated the effects of culture and OLR on changes in mitochondrial dynamics by measuring mitochondrial membrane potential using flow cytometry. Mitochondrial membrane potential (Δψm) is an important indicator of the energy state not only of mitochondria but also of the cell itself. The results of the viability and metabolic activity assays showed the most significant differences at concentrations of 10 and 40 µg/ml; therefore, these concentrations were chosen for further testing. Incubation of splenocytes for 24 h resulted in a significant decrease in mitochondrial dynamics compared to freshly isolated cells (*p* < 0.05). Media supplemented with OLR at a concentration of 10 µg/ml stimulated mitochondrial potential, with a significant change observed in medium A (*p* < 0.001). However, the suppressive effects of cultivation were not attenuated by a higher concentration of oleoresin (40 µg/ml), and a further decrease in mitochondrial membrane potential was observed in medium B. Data are expressed as mean fluorescence intensity (MFI) and are shown in Fig. 7*a* and *b*.

### The effect of culture conditions and *H. pluvialis* oleoresin on the expression of transcription factor Nrf2 and genes of endogenous antioxidant system of splenocytes

**Figure 8.**
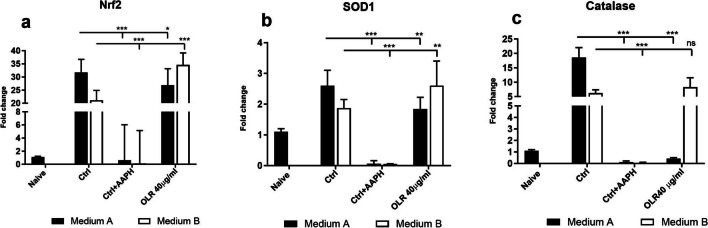
Effect of culture conditions and oleoresin of *H. pluvialis* — OLR (40 µg/ml) on mRNA levels of redox-controlling enzymes (*a*) Nrf2, (*b*) SOD1, and (*c*) catalase of naïve, control cells, control cells after stimulation of radical production by 2,2′-azo-bis-(2-amidinopropane) dihydrochloride (AAPH), and splenocytes in media A and B with different compositions after 24 h. Significant changes in values compared to naïve cells and control group are indicated by *p < 0.05; **p < 0.01; ***p < 0.001.

The influence of the culture conditions and OLR on the expression of the transcription factor Nrf2, as well as genes of the endogenous antioxidant system of cells SOD and catalase, was evaluated. Data from freshly isolated cells were used as a control value for calculating relative gene expression. To determine whether OLR modulates the effects of culture conditions, a gene expression study was performed using 40 µg/ml of this compound (Fig. 8). The mRNA levels for the Nrf2 gene were significantly stimulated after 24 h of culture compared with freshly isolated splenocytes. We observed a significant increase in gene expression for catalase. Culture conditions had the least effect on SOD1 expression. Co-culturing of cells with OLR modulated upregulated gene expression for all three genes compared with control cells. The mRNA transcripts were significantly decreased in cells cultured in medium A, whereas they were increased in cells cultured in medium B. The greatest difference after the addition of OLR was observed for catalase.

### Determination of nitric oxide production in splenic cells

**Figure 9.**
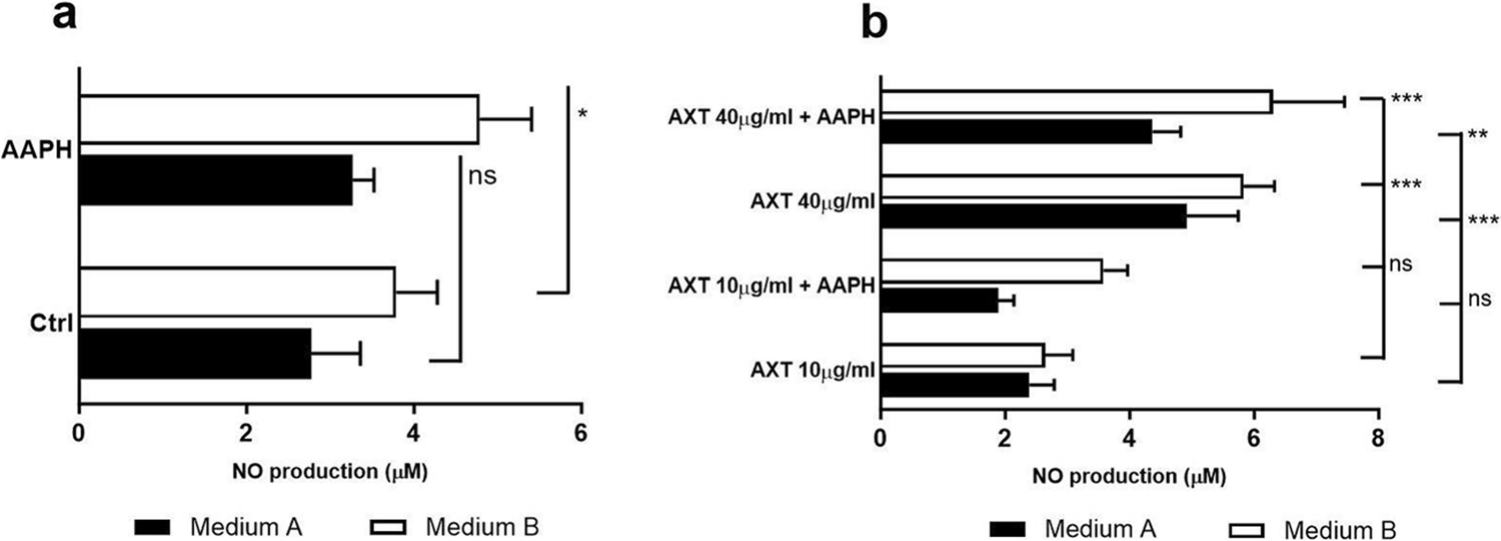
Spontaneous production of nitrite oxide (NO) by untreated splenocytes (Ctrl) and cells treated with 10 µg/ml or 40 µg/ml of oleoresin from *H. pluvialis* (OLR) alone or in combination with AAPH. Cells were incubated with OLR in medium A and B for 24 h and 2,2′-azo-bis-(2-amidinopropane) dihydrochloride (AAPH) was added 1 h before the end of cultivation. Significant changes in values compared to the control group are indicated by **p < 0.01; ***p < 0.001; other values were not significant.

Nitride oxide is a diatomic free radical and is known to play an important role in a wide range of physiological functions, including the immune response. Because of the extremely short physiological half-life of this radical, the production of NO was determined indirectly in the supernatants after culturing of splenocytes by measuring nitrite (NO_2_^−^) with the Griess reagent. As shown in Fig. 9, culturing splenocytes in medium B for 24 h caused a slight increase in NO compared with control cells in medium A, which increased after stimulation by AAPH. Higher concentrations of OLR (40 µg/ml) stimulated the production of NO more than lower concentrations (10 µg/ml). Addition of AAPH stimulated the production of NO at both OLR concentrations, but only in medium B. In contrast, the addition of AAPH in medium A caused a slight decrease in the production of NO at both concentrations.

## Discussion

The selection of an appropriate culture medium and culture conditions for each cell type is extremely important in *in vitro* experiments and affects the significance of the results. Cell cultures are usually operated in an incubator with a precisely controlled temperature, atmospheric air with a volume fraction up to 21% O_2_ enriched with 5% CO_2_, and humidity provided by spontaneous or controlled water evaporation (Cree 2011). In this work, using splenocytes from healthy mice, we investigated the effects of the two most commonly used cell culture media, which differ in the free radical reducing compounds sodium bicarbonate and 2-ME at standard culture temperature, atmospheric oxygen, and 5% CO_2_ content. We also evaluated the antioxidant and cytoprotective effects of astaxanthin-rich oleoresin produced from the microalga *H. pluvialis* on splenocytes grown under both culture conditions. In general, antioxidants absorb free radicals and, through various reactions, ensure their conversion into less reactive and more stable forms.

The results of cell viability assays in two differently composed culture media showed that incubation of splenocytes for 24 h under standard culture conditions had a negative effect on the cells compared with intact, freshly isolated cells, manifested by a decrease in the number of living cells. This was probably caused by incubation of the cells under conditions with non-physiological oxygen levels. We also noted a 30% decrease in the metabolic activity of spleen cells under normal reducing conditions of medium A to a 40% decrease under super-reducing conditions of medium B, as assessed by the MTT assay. We also detected a decrease in viability using the neutral red uptake, which operates on a different principle than the MTT assay. It is one of the most commonly used tests to determine cytotoxicity with many biomedical applications (Repetto *et al.* 2008). In recent years, many studies have shown that standard culture conditions for various cells do not reflect physiological *in vivo* conditions, where organs and tissues are characterized by their own unique “tissue normoxia” or state of physioxia. Physioxia values have been found to range from 1 to 11% O_2_, whereas current *in vitro* experiments with mammalian cell cultures are typically performed at atmospheric O_2_ (18–19.9%) (Alva *et al.* 2022). Thus, cells cultured under standard conditions are indeed exposed to elevated oxygen levels (hyperoxia), which significantly affects the production of ROS (Maddalena *et al. *2017), mitochondrial functions (Moradi *et al.* 2021), and the body’s response to drugs (Fonseca *et al.* 2018). The availability of oxygen also has a significant effect on the metabolism of cultured cells, as they can take up oxygen from solution, and to a lesser extent can affect the expression of genes (Wenger 2000, 2002). When cells are cultured in a hyperoxic environment, there is a decrease in metabolic activity and the formation of reactive oxygen species (Lee and Choi 2003).

To attenuate the effect of free radicals on spleen cells, we examined a concentration-dependent effect of oleoresin on cells cultured under both culture conditions. Most of the oleoresin consists of esterified forms of astaxanthin monoesters (25%) and diesters (75%), and only 5% corresponds to non-esterified astaxanthin (Lorenz and Cysewski 2000; Fabryova *et al.* 2020). Astaxanthin and, to a lesser extent, lutein are known primarily for their significant antioxidant properties. Compared to vitamin E, astaxanthin has 500 times greater antioxidant activity and 38 times greater potential free radical scavenging potential than β-carotene (Oslan *et al.* 2021). It effectively suppresses the action of singlet oxygen; absorbs superoxides, hydrogen peroxides, and hydroxyl radicals; and inhibits lipid peroxidation (Miki 1991; Palozza and Krinsky 1992). Unlike lycopene, β-carotene, and lutein, it has no pro-oxidant effect (McNulty *et al.* 2007). Oleoresin components at tested concentrations up to 10 ug/ml effectively terminated the chains of free radicals generated as a result of hyperoxia, reduced oxidative stress, and thus positively stimulated the viability and metabolic activity of splenocytes under normal reducing conditions of medium A. Medium B additionally contained 2-mercaptoethanol, which was reported to stimulate the survival and growth of lymphocytes cultured *in vitro* (Bannai 1992). Nevertheless, high concentrations of the extract (> 20 µg/ml) showed no beneficial effects on cell viability under these super-reducing conditions of medium B. Several studies have shown that reactive oxygen species act as important physiological regulators of intracellular metabolic pathways (D'Autreaux and Toledano 2007; Finkel 2011; Zhang *et al.* 2016). Redox balance has been shown to be critical for both physiological and pathological processes in cells, as ROS can activate or deactivate various receptors, proteins, ions, and other signalling molecules. When this balance is disturbed by excessive accumulation or on the contrary, by a deficiency of ROS, numerous signalling pathways are affected, which consequently leads to cell dysfunction and the development of pathological conditions.

We hypothesize that the viability of splenocytes was significantly reduced by the oxidative stress that occurs in a hyperoxic environment as a result of improperly adjusted but standard culture conditions. Intact cells after isolation from the spleen produced higher concentrations of ROS compared with cultured cells. We hypothesize that these ROS concentrations are physiologically necessary for cell function. The decrease in production of ROS was likely the result of decreased metabolism in the cells. The addition of OLR to the culture medium multiplied the antioxidant effect and resulted in a significant decrease in reactive molecules, which prevented the necessary signalling between cells. We also confirmed these results by testing the effect of oleoresin on the production of ROS, where treatment with a higher tested concentration in both tested culture media resulted in a significant decrease in the amount of oxygen radicals, even after addition of AAPH.

Changes in mitochondrial dynamics were assessed by changes in mitochondrial membrane potential (MMP; Δψm), and the effects of culture on apoptosis were also assessed because mitochondrial dysfunction and loss of mitochondrial membrane potential are associated with the intrinsic apoptotic pathway (Stahler and Roemer 1998). Consistent with our previous results, a high number of control cells were in an early stage of apoptosis after cultivation, indicating that cultivation conditions affect cell viability. Short-term hyperoxia exposure leads to mitochondrial dysfunction, which is manifested by a decrease in membrane potential (Audi *et al.* 2022; Alva *et al.* 2023). Treatment of cells with oleoresin at concentrations up to 10 µg/ml had a positive effect on the mitochondrial membrane dynamics, and the same trend was confirmed by the effects of oleoresin on the numbers of live/apoptotic cells. However, concentrations of oleoresin > 20 µl/ml probably resulted in an imbalance in mitochondrial homeostasis as well as in increase in the number of cells in the early stages of apoptosis. This effect was stronger under the super-reducing conditions of medium B, whereas the reducing environment in medium A showed a slightly more positive effect on the percentage of live cells/cells in the early stage of apoptosis after the treatment with oleoresin.

To maintain the homeostatic level of ROS, cells employ antioxidant mechanisms, which include antioxidant enzymes (Ma 2013). In our work, cells responded to ex vivo cultivation with the changes in mRNA expression of the transcription factor Nrf2, SOD1, and catalase, which were increased after cultivation. The Nrf2 transcription factor regulates redox homeostasis in cells and is tightly regulated at multiple levels, including gene transcription under the influence of stress factors, which include oxidative stress. Thus, this transcription factor enables the activation of the cellular endogenous antioxidant mechanisms (Hayes and Dinkova-Kostova 2014). Transcription of all monitored genes was upregulated in control cells, indicating changes in redox balance in cells. Addition of oleoresin to the culture media modulated the expression of endogenous antioxidant enzymes in medium A and B, respectively. Xanthophylls, including astaxanthin, are more electrophilic compared to other carotenoids, especially carotenes, which makes them bioactive and thus stimulates the expression of transcription factors to a greater extent (Linnewiel *et al. *2009; Linnewiel-Hermoni *et al.* 2014). Our results showed that carotenoids can also act as direct antioxidants and reduce the levels of peroxyl radicals and singlet oxygen, but their important biological function is primarily to activate the cell’s antioxidant system by regulating the expression of the major redox transcription factor Nrf2. Addition of AAPH to cells almost completely inhibited gene transcription, although maximal stimulation was expected, at least for Nrf2. The inhibitory effect of AAPH may be related to the deleterious effect of the molecule on this biological process. Treatment of cells with oleoresin had a positive effect on mRNA levels for SOD1 and catalase under super-reducing conditions of medium B compared with cells in the control group. The mRNA levels for both tested enzymes were significantly inhibited under normal reducing conditions of medium A compared with the control group, which may be attributed to the gradual induction of redox homeostasis.

Nitric oxide is an important signalling molecule produced by inducible nitric oxide synthase (iNOS) of immune cells, especially macrophages and NK cells, in response to various inflammatory mediators (Diefenbach *et al*. 1998; Xue *et al.* 2018). To a lesser extent, nitric oxide is also produced by the lymphoid cell lineage, which accounts approximately 80% of all cells in the spleen (Palmieri *et al.* 2020). Therefore, we were only able to detect low levels of nitric oxide in our experiments. The results also showed that culture conditions had only a minor effect on the production of NO, and higher production was observed in medium B. The concentration of NO was significantly increased after treatment compared to the control, especially at a lower concentration of oleoresin (10 µg/ml), and more NO was produced by the cells in medium B. Stimulation with AAPH did not further significantly increase the concentration of NO. Ritchie *et al*. (Ritchie *et al.* 2017) showed that the formation of NO can be significantly reduced or directly inactivated by superoxide anions. Increased concentrations of NO produced by lymphocytes after treatment with oleoresin in our work correlate with significantly reduced oxidative stress and ROS concentration, supporting our previous findings.

## Conclusions

The standard culture conditions used: 37°C, with access to ambient air (~ 20% O_2_ content), enriched with 5% CO_2_, were found to be highly hyperoxic, as evidenced by a significant decrease in the number of living cells, a decrease in their metabolic activity, and absorption of nutrients. The negative effects of incubation were accompanied by damage to mitochondria and transition of cells to the early stage of apoptosis due to increased oxidative stress after 24 h of incubation. High levels of ROS/RNS also activated the antioxidant defence mechanisms of splenocytes via the signalling Nrf2 pathway. A stronger negative effect on cells after 24 h of incubation was observed under the super-reducing conditions of medium B, in which there was an extreme reduction in free radical content. However, maintenance of a basal level of ROS/RNS is essential for proper cell physiology. The negative effects of culturing were mitigated by treating cells with a lower tested concentration (10 µg/ml) of oleoresin derived from the microalga *H. pluvialis*, which showed significant concentration-dependent cytoprotective and antioxidant effects on spleen cells of Balb/c mice.

### Supplementary Information

Below is the link to the electronic supplementary material.

Supplementary file1 (JPG 30 KB) Fig. 1 HPLC analysis of *H. pluvialis oleoresin*

## Data Availability

Data supporting this study are included within the article and supporting materials.
