# Supplementary figures and images for: Influence of standard culture conditions and effect of oleoresin from the microalga Haematococcus pluvialis on splenic cells from healthy Balb/c mice — a pilot study
Source: In Vitro Cell Dev Biol Anim. 2023 Dec 7;59(10):764–77. doi: 10.1007/s11626-023-00822-x (PMC10739404; doi:10.1007/s11626-023-00822-x)

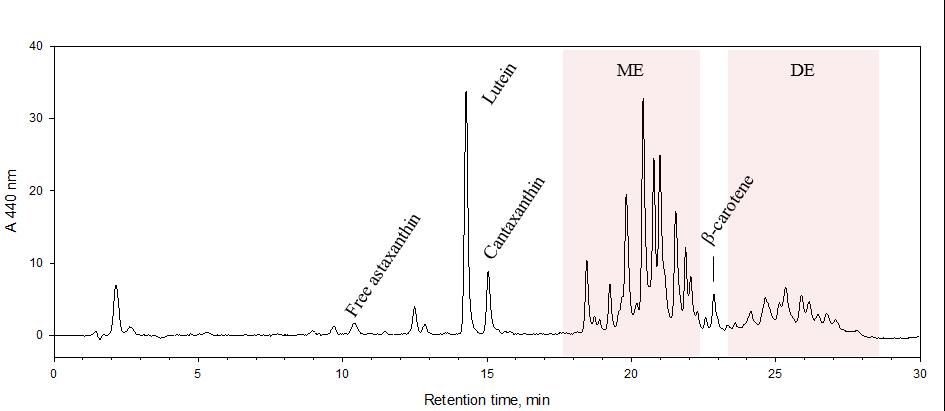

Supplement: Supplementary file 1 — Supplementary file1 (JPG 30 KB) Fig. 1 HPLC analysis of H. pluvialis oleoresin [file 11626_2023_822_MOESM1_ESM.jpg]
